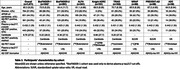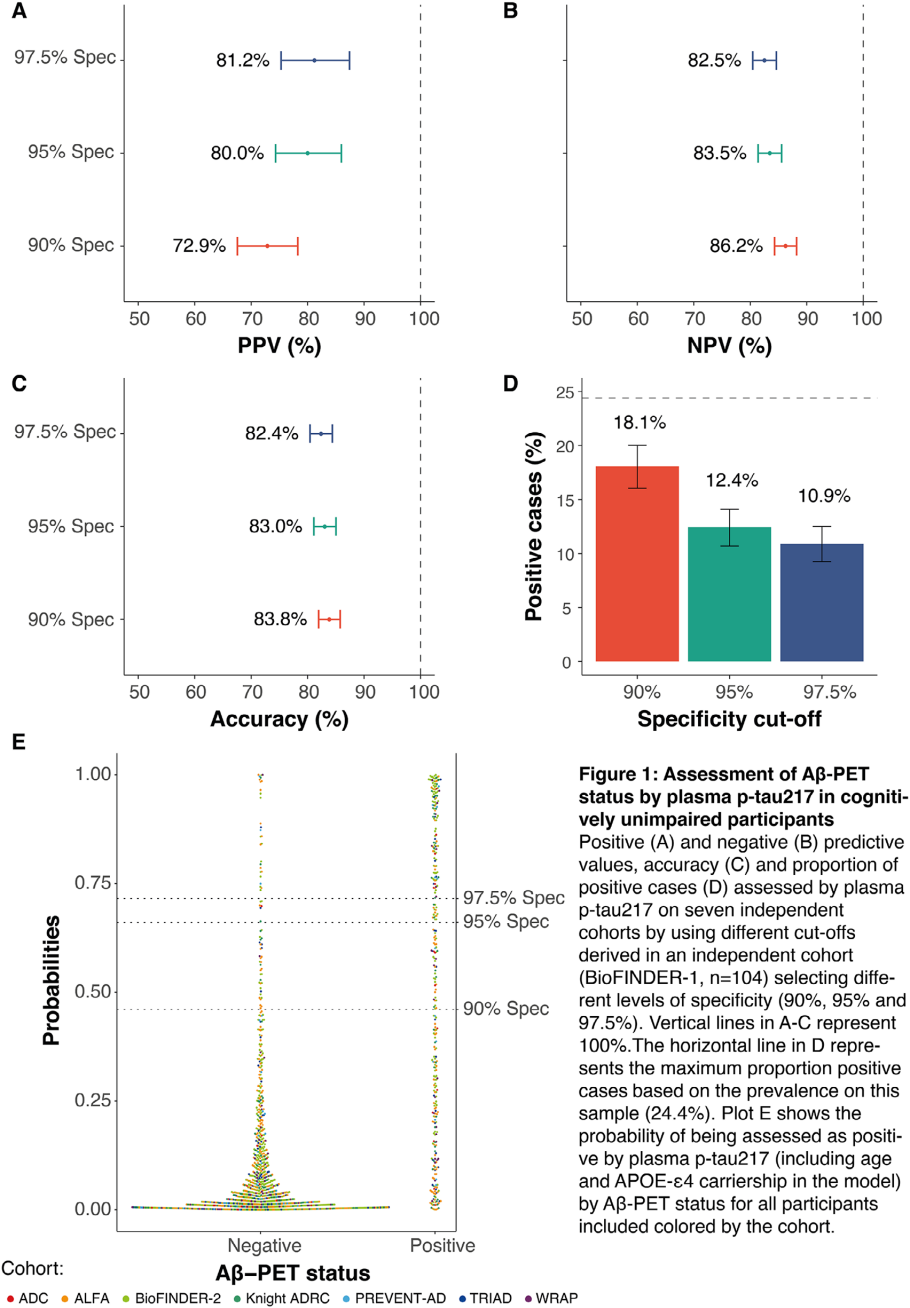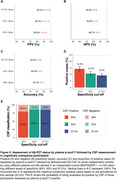# Use of plasma p‐tau217 as a pre‐screening method for detecting amyloid‐PET positivity in cognitively unimpaired participants: A multicenter study

**DOI:** 10.1002/alz.085773

**Published:** 2025-01-09

**Authors:** Gemma Salvadó, Shorena Janelidze, Joseph Therriault, Wagner Scheeren Brum, Alexa Pichet Binette, Erik Stomrud, Niklas Mattsson‐Carlgren, Sebastian Palmqvist, Tammie L.S. Benzinger, Juan Domingo Gispert, Kaj Blennow, Sylvia Villeneuve, Sterling C. Johnson, Pedro Rosa‐Neto, Suzanne E. Schindler, Marc Suarez‐Calvet, Rik Ossenkoppele, Oskar Hansson

**Affiliations:** ^1^ Clinical Memory Research Unit, Department of Clinical Sciences, Lund University, Lund Sweden; ^2^ Translational Neuroimaging Laboratory, The McGill University Research Centre for Studies in Aging, Montréal, QC Canada; ^3^ McGill University, Montreal, QC Canada; ^4^ Department of Psychiatry and Neurochemistry, Institute of Neuroscience and Physiology, The Sahlgrenska Academy, University of Gothenburg, Mölndal Sweden; ^5^ Graduate Program in Biological Sciences: Biochemistry, Universidade Federal do Rio Grande do Sul (UFRGS), Porto Alegre Brazil; ^6^ Memory Clinic, Skåne University Hospital, Malmö Sweden; ^7^ Clinical Memory Research Unit, Department of Clinical Sciences Malmö, Faculty of Medicine, Lund University, Lund Sweden; ^8^ Wallenberg Center for Molecular Medicine, Lund University, Lund Sweden; ^9^ Washington University School of Medicine in St. Louis, St. Louis, MO USA; ^10^ Knight Alzheimer Disease Research Center, St. Louis, MO USA; ^11^ Centro de Investigación Biomédica en Red Bioingeniería, Biomateriales y Nanomedicina (CIBER‐BBN), Instituto de Salud Carlos III, Madrid Spain; ^12^ Hospital del Mar Research Institute, Barcelona, Barcelona Spain; ^13^ Universitat Pompeu Fabra, Barcelona Spain; ^14^ Barcelonaβeta Brain Research Center (BBRC), Pasqual Maragall Foundation, Barcelona Spain; ^15^ Centro Nacional de Investigaciones Cardiovasculares (CNIC), Madrid Spain; ^16^ Clinical Neurochemistry Laboratory Sahlgrenska University Hospital, Mölndal Sweden; ^17^ Institute of Neuroscience and Physiology Sahlgrenska Academy at the University of Gothenburg, Gothenburg Sweden; ^18^ Douglas Mental Health University Institute, Centre for Studies on the Prevention of Alzheimer's Disease (StoP‐AD), Montréal, QC Canada; ^19^ Douglas Mental Health Research Centre, Montreal, QC Canada; ^20^ Wisconsin Alzheimer’s Disease Research Center, University of Wisconsin‐Madison School of Medicine and Public Health, Madison, WI USA; ^21^ Wisconsin Alzheimer's Institute, University of Wisconsin School of Medicine and Public Health, Madison, WI USA; ^22^ Centre for Studies on Prevention of Alzheimer's disease (StoP‐AD Centre), Montreal, QC Canada; ^23^ Washington University in St. Louis School of Medicine, St. Louis, MO USA; ^24^ Hospital del Mar Research Institute (IMIM), Barcelona Spain; ^25^ Centro de Investigación Biomédica en Red de Fragilidad y Envejecimiento Saludable (CIBERFES), Instituto de Salud Carlos III, Madrid Spain; ^26^ Servei de Neurologia, Hospital del Mar, Barcelona Spain; ^27^ Alzheimer Center Amsterdam, Amsterdam UMC, Amsterdam Netherlands; ^28^ Clinical Memory Research Unit, Lund University, Lund Sweden; ^29^ Amsterdam Neuroscience, Neurodegeneration, Vrije Universiteit Amsterdam, Amsterdam Netherlands

## Abstract

**Background:**

Recent results from clinical trials in Alzheimer’s disease (AD) emphasize the importance of treating early‐stage disease. However, recruitment of preclinical AD participants is difficult due to the lack of symptoms, and the costs and/or invasiveness of established CSF and PET tests. We aimed to investigate whether plasma p‐tau217 could be used to pre‐screen cognitively unimpaired (CU) potential participants for amyloid‐β (Aβ) pathology to improve the efficiency of clinical trial recruitment.

**Method:**

We included 1,471 CU participants from eight cohorts (Table 1) with available plasma p‐tau217, Aβ CSF biomarkers and Aβ‐PET status (served as standard‐of‐truth). Plasma p‐tau217 concentrations were z‐scored based on Aβ‐negative participants and harmonized across cohorts using neuroCombat. Cut‐offs for plasma p‐tau217 were derived in the BioFINDER‐1 cohort (n=104) based on different specificity levels (90%, 95% and 97.5%) to maximize positive predictive values (PPV). These cut‐offs were used in the other cohorts to assess the accuracy of plasma p‐tau217 for detecting Aβ‐PET positivity. Next, within plasma positive participants only, we evaluated the value of dichotomized Aβ CSF (based on established clinical thresholds) on assessing Aβ‐PET positivity. All models included age and APOE‐ε4 carriership as covariates.

**Result:**

334 (24.4%) of participants were Aβ‐PET positive. Using the a priori defined cutoffs, plasma p‐tau217 categorization resulted in high PPVs (72.9%‐81.2%), negative predictive values (NPVs, 82.5%‐86.2%), and accuracy (82.4%‐83.8%), with an overall rate of positivity between 10.9%‐18.1% (Figure 1). When applying CSF biomarkers to the plasma positive participants in a second step, the PPVs increased up to 90.8%‐95.3%, with NPVs ranging between 82.8%‐86.7% and accuracies between 84.0%‐87.3%, with a slight decrease in the proportion of overall positive cases (9.3%‐14.3% from the original sample) given that the CSF positivity in the plasma positive participants ranged between 79.4%‐85.2% (Figure 2).

**Conclusion:**

Plasma p‐tau217 can identify Aβ‐PET positive CU individuals with PPVs reaching 81%, which can be further improved to PPVs of up to 95% with a subsequent CSF measurement. Plasma p‐tau217 could be used, either as stand‐alone biomarker, or as an initial step before CSF biomarkers (reducing their need by ∼80‐90%), for pre‐screening in clinical trials of preclinical AD depending on the certainty needed for Aβ‐PET positivity.